# Can Non-Invasive Spectrophotometric Hemoglobin Replace Laboratory Hemoglobin Concentrations for Preoperative Anemia Screening? A Diagnostic Test Accuracy Study

**DOI:** 10.3390/jcm12175733

**Published:** 2023-09-03

**Authors:** Maryam Alwabari, Fatimah Alhamad, Fatimah Alsahaf, Fatima Al Amer, Fatma Alniniya, Imran Alherz, Nawal Omer, Abdulaziz Bushehab, Khaled Yassen

**Affiliations:** 1 Anesthesia Department, King Fahad Hospital, Ministry of Health, Hofuf 36441, Al Ahsa, Saudi Arabia; alwabari.maryam.e@gmail.com (M.A.); majbourha@gmail.com (F.A.); dr.imran.alherz@gmail.com (I.A.); 2 College of Medicine, King Faisal University, Hofuf 31983, Al Ahsa, Saudi Arabia; fatma19992013@gmail.com (F.A.); fatimah.sahhaf@gmail.com (F.A.); fatima.alamer595@gmail.com (F.A.A.); 3 Hereditary Blood Disease Center, Hofuf 36422, Al Ahsa, Saudi Arabia; gelas3@hotmail.com; 4 Nursing Services, Hereditary Blood Disease Center, Hofuf 36422, Al Ahsa, Saudi Arabia; abdulazizbushehab@gmail.com; 5 Anesthesia Unit, Surgery Department, College of Medicine, King Faisal University, Hofuf 31983, Al Ahsa, Saudi Arabia

**Keywords:** hemoglobin, anemia, monitoring, non-invasive, preoperative

## Abstract

Preoperative assessment of hemoglobin concentration in blood is important to diagnose anemia. The primary aim of this prospective diagnostic test accuracy study was to monitor non-invasive spectrophotometric hemoglobin (SpHb, g/dL) concentrations among adults prior to elective surgery and to investigate the correlation and agreement of SpHb with laboratory hemoglobin (Hb, g/dl). A secondary aim was to identify the anemia cut-off values for SpHb based on the World Health Organization (WHO) definitions for anemia. This study included 151 consecutive patients (age ≥ 18 year) presenting for preoperative evaluation prior to scheduled elective general or orthopedic surgery. Results identified the mean ± SD of SpHb at 11.43 ± 2.01 g/dL, which underestimated the mean laboratory Hb (12.64 ± 2.29 g/dL, *p* < 0.001). A bias mean difference (SpHb–Hb) of −1.21 g/dL, with a SD of 1.76, was reported. This bias (SpHb–Hb) was inversely correlated with the mean Hb concentrations. A positive correlation existed between SpHb and Hb, with a good degree of reliability and a significant Intra Class Correlation (ICC). SpHb diagnosed anemia in 32.3% and 60.3% of males and females, respectively. The SpHb cut-off values to identify anemia were 11.3 and 10.2 g/dL for males and females, respectively, with a sensitivity of 83.3% for males and only 62.9% for females. The specificity for males and females were 81% and 91.3%, respectively. SpHb sensitivity allows for anemia diagnosis among males, but not females. However, the specificity allows SpHb to rule out anemia for both.

## 1. Introduction

Preoperative anemia management is one of the known patient blood management (PBM) pillars [1]. Improving blood hemoglobin concentrations prior to elective surgery is essential to reduce perioperative morbidity and mortality [2,3,4,5]. Sickle cell disease, thalassemia, and other types of hemoglobin disorders are present in Saudi Arabia, particularly in the Eastern Province of the Kingdom, where this current study population lives. Anemia can exist in various forms and can be discovered accidentally prior to surgery [6,7,8].

An anesthesiologist frequently depends on the hemoglobin concentrations measured by the laboratory complete blood count (CBC) to rule out anemia and related diseases during pre-anesthesia clinics visits. The presence of a non-invasive method will additionally reduce the discomfort of venipunctures, laboratory costs, and time. SpHb is a safe and portable method to measure hemoglobin concentrations based on optics. Anemia prior to surgical procedures varies greatly depending on associated diseases and the study population. The sensitivity and specificity of the non-invasive measurement of spectrophotometric hemoglobin concentration (SpHb) in comparison to the gold standard measurement of hemoglobin (Hb by the laboratory hematology cell analyzer) vary across different studies. Previous studies have indicated that SpHb has poor precision, particularly when peripheral perfusion is low. The performance and accuracy of several non-invasive hemoglobin devices available on the market are still being debated. Khalafallah et al., in their RCT (2015), reported an increase in the SpHb accuracy (sensitivity) at low Hb values for males compared to females. Honnef et al. (2022), in an observational study, reported that the sensitivity of SpHb to identify anemia was low, in contrast to the prospective study by Wittenmeier et al. (2021) [9,10,11]. This diversity between various researchers motivated our team to design this current research to study the Masimo Radical-7 performance as a non-invasive hemoglobin monitor. This device utilizes multiple wavelengths of light (500–1300 nm) to measure the fractional hemoglobin and calculate the total hemoglobin concentration [12,13]. The primary aim of this study was to measure the non-invasive SpHb (g/dL) among adults prior to surgery and to investigate SpHb correlation and agreement with laboratory hemoglobin (Hb, g/dl), which was measured within 24 h of planned anesthesia and surgery. A secondary aim was to identify the SpHb cut-off values for anemia among the study population based on the World Health Organization (WHO) definitions for anemia [14]. Finally, the aim was to report any factors that affect SpHb accuracy.

## 2. Materials and Methods

A prospective diagnostic test accuracy study was approved by the local ethics committee, Al-Ahsa Health Cluster (IRB KFHH No H-05-HS-065), IRB Log No: 08-EP-2023, Saudi Arabia.

### 2.1. Patients

A single center study was conducted at King Fahad Hospital Hofuf (KFHH), Hofuf, Al Ahsa, Saudi Arabia, between 26 January and 1 May 2023. The demographics, American Society of Anesthesiology (ASA) classification, and laboratory data of the 151 consecutive patients were reported. This study included adult (18–60 year) patients with ASA grades from 1 to 3. All consecutive patients (age ≥ 18 years) presenting for preoperative evaluation prior to elective general or orthopedic surgery were included. Exclusion criteria included a refusal to consent, severe disease with a constant threat to life (ASA > 4), and patients with a recent history of hemorrhagic surgery. Others exclusion criteria included vascular surgery, pediatric surgery, hemodynamically unstable patients, and those admitted following major trauma with blood loss, missing data, and hepatic and jaundiced patients. Elective general and orthopedic surgery were only included to avoid the reported effects of specific types of surgery on the SpHb values [12,15].

### 2.2. Hemoglobin, Perfusion Index, and Pleth Variability Index Measurements

SpHb (g/dL), the Pleth Variability Index (PVI, %) and the Perfusion Index (PI) were measured via finger probe sensors (Masimo Radical-7, Irvine, CA, USA). The Masimo Radical-7 pulse co-oximeter calculates the hemoglobin concentration (SpHb) non-invasively using transcutaneous spectrophotometry-based technology. The Rainbow signal extraction technology or the spectrophotometric estimation of hemoglobin (SpHb) is the technology studied. It overcame invasive laboratory Hb venous sampling by enabling instant, rapid, needle-free hemoglobin measurement. The figure sensors were covered with an optical shield to protect against light interference. SpHb and the Perfusion Index (PI) were recorded only when patients were at rest, to minimize movement interference.

This device utilizes a multi-wavelength analysis to measure Hb absorption and calculate total Hb concentrations (SpHb). Hemoglobin is known as a dominant absorber in blood at 575–1100 nm.

PVI is another measurement that automatically and continuously calculates the respiratory variations in the photoplethysmosgraphic waveform (pleth). In other words, PVI is a continuous non-invasive parameter that demonstrates the variability in the pleth during respiratory cycles. PVI is a dynamic indicator of fluid responsiveness with a normal range between 9 and 13%.

The third parameter measured was the PI, which is the ratio of the pulsatile blood flow to the non-pulsatile static blood flow in a patient’s fingertip. The Perfusion Index is an indication of the pulse strength at the sensor site. The PI’s values are known to range from 0.02% for a very weak pulse to 20% for an extremely strong pulse. A higher Perfusion Index means greater blood flow to the finger and a lower Perfusion Index means lower blood flow to the finger. The PI relatively assesses pulse strength and perfusion at the finger. A PI value of >2% confirms an adequate signal.

The PI is calculated by indexing the infrared (IR) pulsatile signal against the non-pulsatile signal and expressing this number as a percentage, while the PVI is a measure of the dynamic changes in the Perfusion Index (PI) that occur during one or more complete respiratory cycles. The PI reflects the amplitude of the pulse oximeter waveform and is calculated as the pulsatile infrared signal (AC or variable component), indexed against the non-pulsatile infrared signal (DC or constant component). AC represents the variable absorption of infrared light due to pulsating arterial inflow and DC represents the constant absorption of infrared light due to skin and other tissues. The PI = AC/DC × 100% and by using the PI, the PVI is calculated as PVI = PImax – PImin/PImax × 100%.

The PVI was displayed as a percentage (numerical value) and a trend graph. The lower the PVI value, the less variability there is in the PI over a respiratory cycle. The higher the variability, the more likely the patient will respond to fluid infusion with an increase in cardiac output.

A period of five minutes was allowed prior to recording the SpHb readings to allow for stability and reduce movement interference. SpHb was measured by the Radical-7 device from Masimo Corporation, Irvine, CA, USA, with the latest software version. The disposable Rainbow probe sensor for SpHb measurement was placed on the fifth finger. The non-invasive blood pressure cuff or pulse oximeter probes were not placed on the same arm or hand where SpHb was measured. Only one senior registered nurse in the operating room performed all SpHb measurements. The sensor was placed on the patient’s ring finger, while the site of phlebotomy was in the opposite hand. The device requires time for self-calibration and this was monitored through a visual graph and a sound. This process of self-calibration can take approximately 1 min. In case of any failure, a second attempt was performed, this time using the middle finger on the same hand or the ring finger on the opposite hand as alternatives.

The Perfusion Index (PI) reflects the extent of peripheral blood flow circulation in the finger. According to the manufacturer’s recommendation, the lowest PI allowed is 0.3, with a PI above 0.3 necessary to allow for a reliable SpHb measurement. If no or poor signal was recorded, or if the PI was low, the alternative hand was used instead. Patients with no device response or with a poor signal were excluded. 

Hemoglobin concentrations from the CBC were considered as the gold standard reference for true hemoglobin concentrations. All laboratory hemoglobin was retrieved from the complete blood count analysis performed via venous blood collection within 24 h of SpHb measurement. All venous blood samplings were performed by phlebotomy professionals. Laboratory hemoglobin was measured by the Abbott CELL-DYN Ruby hematology analyzer (Abbott, Abbott Park, IL, USA). Two mL ethylenediaminetera acetic acid blood specimens in an (K2EDTA) test tube (BD Vacutainer, Becton Dickinson, Franklin Lakes, NJ, USA) were thoroughly mixed and then immediately transferred to the central laboratory for analysis by a hematology analyzer. The hematology analyzer measures hemoglobin by colorimetry and utilizes the cyanide-free, sodium lauryl sulfate method. The oxygen saturation percentage (%) was measured with the Masimo Radical-7 and was reported in the results. A recent report by Gomaa et al. reported that oxygen saturation affects the SpHb accuracy [16].

Hb concentrations, both high and low, were reported and the effect of the PI and Pleth Variability Index (PVI, %) were studied statistically, as they may have an impact on the accuracy of the non-invasive SpHb measurement.

The World Health Organization (WHO) defines anemia as any Hb concentration below 13 g/dL for males and below 12 g/dL for females [14]. This WHO definition was applied to diagnose anemia in our study.

Measurement time: Immediately prior to surgery.

### 2.3. Statistical Analysis

Data were collected and entered into the computer using the SPSS (Statistical Package for Social Science) program for statistical analysis (ver. 21) [14,17]. Data were entered as numerical or categorical, as appropriate. The Kolmogorov–Smirnov test of normality revealed significance in the distribution of some of the variables, so non-parametric statistics were adopted [18]. Data were described using minimum, maximum, mean, standard deviation, and 95% CI of the mean, median, and inter-quartile range, as appropriate. Categorical variables were described using frequency and percentage. Comparisons were carried out between two studied independent non-normally distributed subgroups using the Mann–Whitney U test [19]. Non-parametric Kendall’s tau correlation (τ) was used. The rule of thumb for interpreting the size of a correlation coefficient and the subsequent interpretation was applied: 0.90 to 1.00 (−0.90 to −1.00) very high positive (negative) correlation; 0.70 to 0.90 (−0.70 to −0.90) high positive (negative) correlation; 0.50 to 0.70 (−0.50 to −0.70) moderate positive (negative) correlation; 0.30 to 0.50 (−0.30 to −0.50) low positive (negative) correlation; 0.00 to 0.30 (0.00 to −0.30) negligible correlation [20]. Intra Class Correlation (ICC) was used to assess agreement. A value less than 0.40 was considered poor; 0.40–0.59 fair; 0.60–0.74 good; 0.75–1.00 excellent [21]. A Bland–Altman assessment of the two assay methods, as recommended by Mantha and colleagues, was used [22]. An evaluation of ICC coefficient values using Cicchetti guidelines was adopted [23].

The minimal sample size was calculated based on a previous study by Okazaki et al., which aimed to estimate the accuracy of a non-invasive Hb monitor in this age group [24]. Okazaki et al. (2022) concluded that non-invasive Hb can be used to evaluate Hb levels among school children for health promotion or research purposes because of its extremely low bias (or precision) and no systematic biases (including fixed or proportion biases), and a positive correlation existed between non-invasive monitoring and blood drawing [24]. The sample size was calculated to detect the diagnostic accuracy of non-invasive, spectrophotometric hemoglobin (SpHb) in patients with anemia [25]. Based on Okazaki et al. (2022), a sample size of 59 patients is sufficient to conduct this agreement study, with a significance level of 5% (α error accepted = 0.05) and statistical power (1 − β) of 80% [26]. Any withdrawals from the study were replaced in order to maintain a minimum sample size [27].

## 3. Results

**Figure 1:**
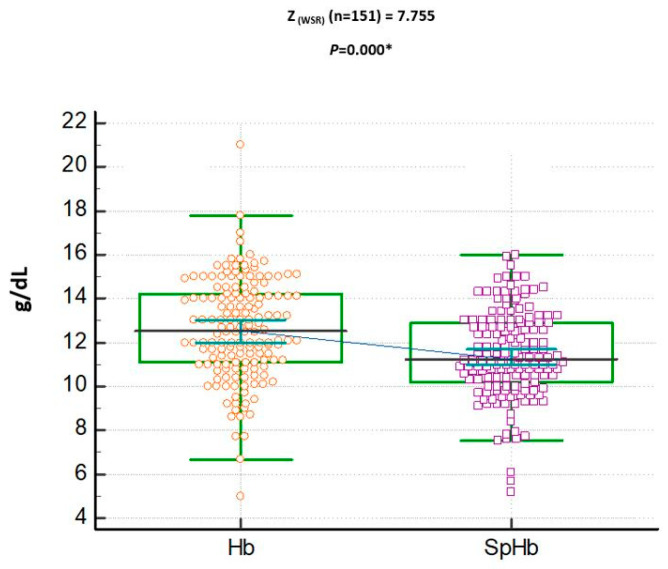
Dot plots with connecting lines for median and 95% confidence interval (CI) of both spectrophotometric hemoglobin (SpHb, g/dL) and laboratory hemoglobin (Hb, g/dL). SpHb underestimated Hb concentration values. WSR: Wilcoxon Signed Rank test performed.* denotes statistical significance.

A total of 156 patient were enrolled, but only 151 patients were included, as 3 patients with poor PI signals (<0.3) and 2 patients with cardiac arrhythmia were excluded. Non-invasive spectrophotometric hemoglobin measurement was not reliable in 5 patients; however, it was successful in 151 of the 156 patients enrolled in this study (failure rate 3.2%). No adverse events were reported while performing SpHb or during the laboratory Hb measurements. Demographics, ASA classification (ASA 1–3), hemoglobin concentrations (g/dL), PVI (%), PI, and oxygen saturation percentage were presented in Table 1. The mean ± SD of SpHb was 11.43 + 2.01 g/dL, which underestimated the mean laboratory Hb of 12.64 ± 2.29 g/dL, *p* < 0.001 (see Figure 1).

**Figure 2:**
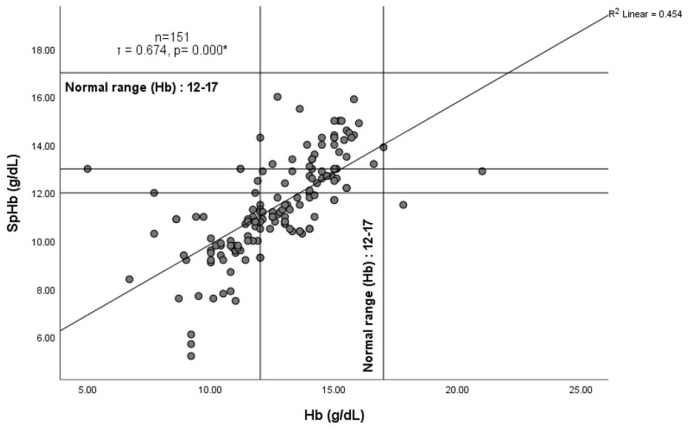
Simple scatter diagram with regression (best fit) line showing a moderate positive correlation between spectrophotometric hemoglobin (SpHb, g/dL) and laboratory hemoglobin (Hb, g/dL). * denotes statistical significance.

**Figure 3:**
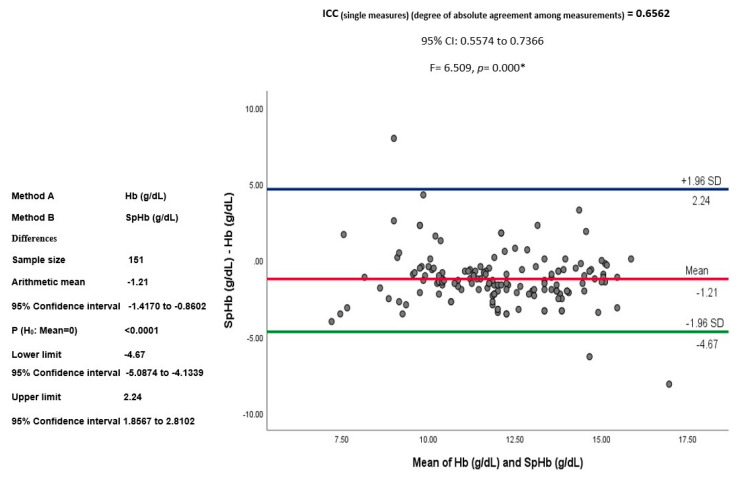
Agreement between spectrophotometric hemoglobin (SpHb, g/dL) and laboratory hemoglobin (Hb, g/dL) demonstrated by Intra Class Correlation (ICC).

A positive correlation existed between SpHb and Hb. Kendall’s tau correlation (τ) was calculated (*n* = 151, τ = 0.674, *p*< 0.001). This was represented in Figure 2 by a simple scatter diagram, with a regression (best fit) line that demonstrated a moderate positive correlation between the spectrophotometric hemoglobin (SpHb, g/dl) and laboratory hemoglobin (Hb, g/dL). The mean difference or bias of SpHb–Hb was −1.21 (SD 1.76) g/dL, with a 95% confidence interval (CI) from −1.41 to −0.86 and a limit of agreement from −4.6 to 2.24 at ±1.96 SD. A good degree of reliability was observed between the bias (SpHb–Hb) and laboratory Hb (g/dL), with an Intra Class Correlation (ICC) of 0.656 and a 95% CI from 0.557 to 0.736 (F = 6.509, *p* = 0.000) (see Figure 3).

**Figure 4:**
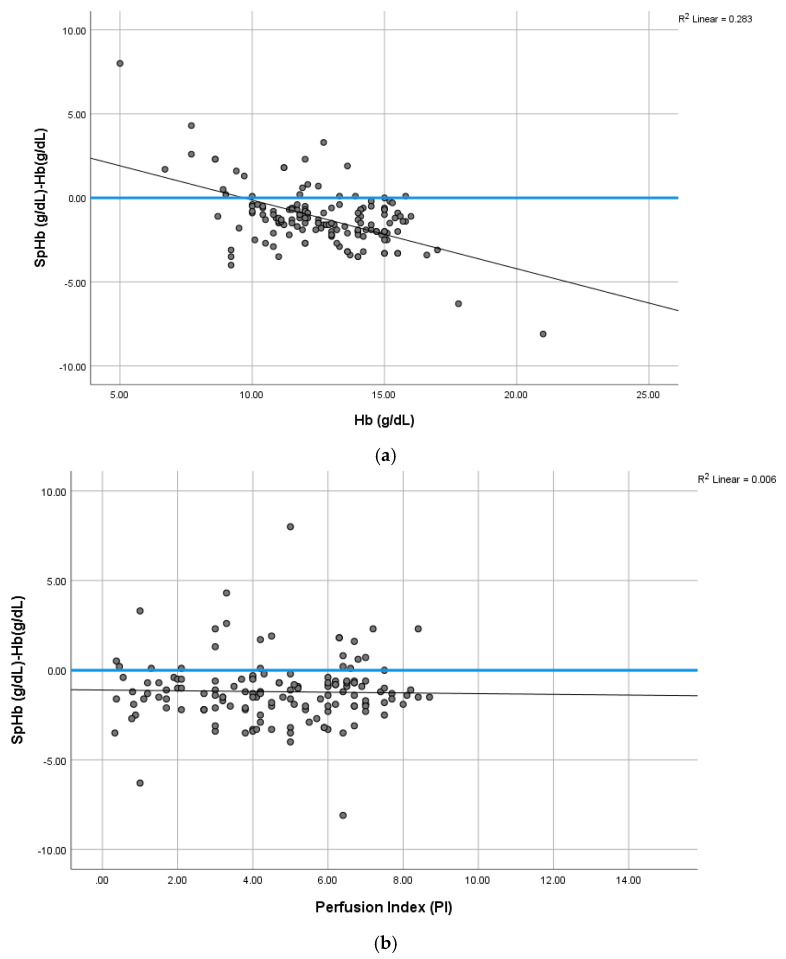
(**a**) Mean difference between SpHb and Hb (g/dL) varies from positive to negative depending on Hb concentration (g/dL). There is a negative difference (underestimate) at high Hb and positive difference (overestimate) at low Hb concentration. The bias is inversely correlated with higher Hb. The trend is illustrated by a non-parametric smoothed regression line. (**b**) The relationship between bias (SpHb–tHb) and Perfusion Index (PI). The trend is illustrated by a non-parametric smoothed regression line.

**Figure 5:**
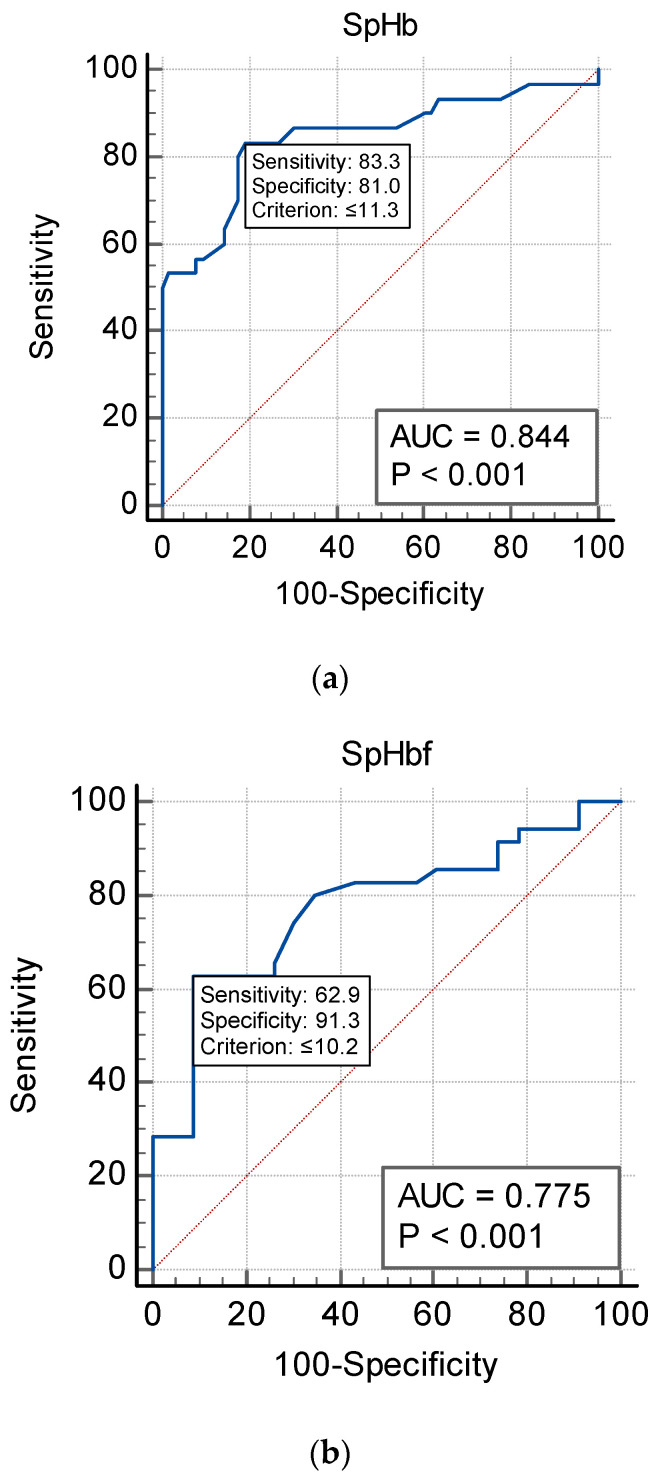

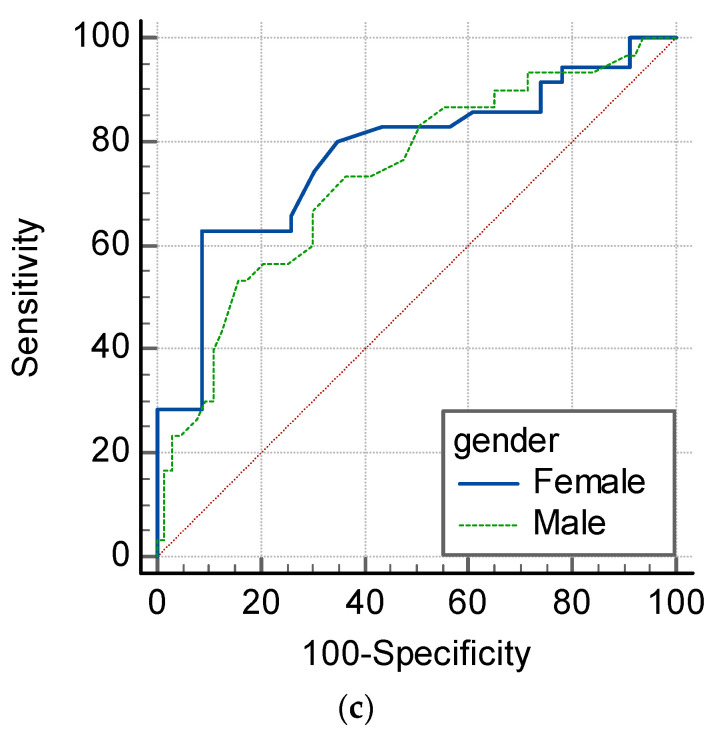
(**a**) Males non-invasive hemoglobin (SpHb) ROC graph demonstrating the ability of SpHb < 11.3 g/dL to diagnose anemia with a sensitivity of 83.3% and specificity of 81.0%. Prevalence of anemic among males = 32.3%. (**b**) Females non-invasive hemoglobin (SpHb) ROC graph demonstrating the ability of SpHb < 10.2 g/dL to diagnose anemia with a sensitivity of 62.9% and specificity of 91.3%. Prevalence of anemic among females = 60.3%. (**c**) Comparison of independent ROC curves for both females and males’ ROC graphs (*p* = 0.61).

The mean bias (SpHb–Hb) was inversely correlated with laboratory Hb concentrations but was not affected by the changes in the PI. At low Hb concentrations (<10 g/dL), SpHb values overestimated laboratory Hb and vice versa at higher Hb concentrations. This was illustrated by the non-parametric smoothed regression line demonstrated in Figure 4a,b. The current study included patients from the Al Ahsa region in the Eastern Province of Saudi Arabia. Table 2 reported the corresponding SpHb cut-off values to identify anemia in this study sample according to the WHO definition. SpHb was able to diagnose anemia in 32.3% of males and 60.3% of females scheduled for elective surgery. None of the included patients with anemia had any hereditary hemoglobin disorder. The SpHb sensitivity was 83.3% for males, but only 62.9% in females. However, the SpHb specificity was 81% and 91.3% for males and females, respectively, as shown in Figure 5a–c and Table 2. The PVI correlated negatively with both SpHb and Hb (r = −0.312, *p* < 0.001 and r = −0.239, *p* = 0.003), respectively. No significant correlations existed between the PI and both SpHb and Hb (r = 0.02, *p* = 0.80 and 0.07, *p* = 0.33, respectively).

## 4. Discussion

SpHb underestimated the mean laboratory Hb with a mean difference (bias, SpHb–Hb) of −1.21 g/dL, (SD 1.76). This bias was inversely correlated with laboratory Hb concentrations. SpHb correlated and agreed with laboratory Hb with a good degree of reliability. The identified SpHb cut-off values for anemia were 11.3 g/dL and 10.2 g/dL for males and females. The instant SpHb measurement precision based on sensitivity was lower than expected. However, the specificity for both males and females were 81% and 91.3%, respectively. Based on these findings, SpHb can rule out anemia among both males and females, but only diagnose anemia among males. The results support the fact that SpHb can swiftly screen and identify anemic patients in outpatient clinics who may require further therapeutic or diagnostic interventions, but SpHb cannot replace laboratory-measured hemoglobin, which is still considered as the gold standard for hemoglobin concentrations in blood and the reference standard recommended by health authorities worldwide.

Non-invasive spectrophotometric hemoglobin measurement was only readable for 151 of the 156 patients included in this study, because of the arrhythmia and low PI effects (failure rate of 3.20%). This failure percentage was similar to the 2% reported by Czempik et al. in their cohort study (2022) and the 5% in Frasca et al.’s prospective study (2011). Both studies retrieved data from patients prior to surgery, as in our current study [28,29]. Hornedo-González et al. also reported no SpHb readings in 10 of the 122 patients in their study [30].

### 4.1. SpHb Agreement with CBC Total Hemoglobin

A moderate degree of correlation and agreement existed between SpHb and laboratory Hb. This represented a significant data analysis outcome. The mean difference bias (SpHb–Hb) reported was −1.21 ± 1.76 (underestimating) with a limit of agreement (LOA) between –4.6 and 2.24 with ±1.96 SD. This wide bias affected the accuracy. Other research teams reported variable results. In Frasca et al.’s study, the bias (SpHb–Hb) was 0.0 and LOA −0.9 to 1 g/dL [29]. Gayat et al. reported a mean bias of 0.56 g.L^−1^ (95% CI 0.41 to 0.69) with an Intra Class Correlation (ICC) coefficient of 0.80 (95% CI 0.74 to 0.84) [31]. Most studies reported the bias difference (SpHb–Hb) within the range of 1–2 g/dL above or below, which is not different from the −1.21 ± 1.76 g/dL bias reported in our current study [11,32,33,34,35]. The ICC in our study was 0.656 with a 95% CI from 0.557 to 0.736, which was again not far from that reported by Gayat et al.

### 4.2. Total Hemoglobin (Hb) and SpHb–Hb Relationship

At extreme concentrations of laboratory hemoglobin, the bias difference was negative at low hemoglobin concentrations and positive at higher concentrations. In other words, the (SpHb–Hb) bias was inversely correlated with the mean laboratory Hb concentrations. This was similar to that reported by Gayat et al. with the Pronto-7 monitor (version 2.1.9, Masimo Corporation, Irvine, CA, USA), which was performed among emergency department patients [31]. The SpHb values in our current study underestimated laboratory hemoglobin. These findings were similar to those presented by Vos et al. in their RCT in 2012 and Miller et al. (2011). Miller et al. reported an inversely correlated relationship between SpHb and Hb concentrations [36,37]. SpHb at a low laboratory hemoglobin concentration can lead clinicians to believe that a patient’s hemoglobin level is normal, when in fact he/she is anemic and a transfusion is required [5].

### 4.3. SpHb Monitoring and Anemia Screening

Khalafallah et al. performed SpHb measurements for 726 patients, with Pronto-7, and compared their results with laboratory Hb using an automated analyzer preoperative and in clinics [9]. They reported that SpHb tended to underestimate the laboratory Hb measurements (negative bias), similarly to the current study. Their sensitivity to detect true anemia by measuring SpHb < 12 g/dL was higher than our study in females (75% vs. 62.9% sensitivity) but closer among males (93% vs. 83.3%). The World Health Organization (WHO) defined anemia as laboratory Hb below 13 g/dL for males and below 12 g/dL for females. In Western countries, anemia is mainly due to the increase in the number of elderly people, nutritional deficiency, or chronic disease. However, in many clinical circumstances, reasons remain unexplained [14]. Our current study included patients from the Al Ahsa region, in the Saudi Arabia Eastern Province known for its prevalence of sickle cell disease and anemia. The SpHb cut-off values identified anemia in 32.3% and 60.3% of males and females, respectively, in our study. There is a need to reduce the number of anemic patients booked for elective surgery.

In the current study, the identified cut-off value for SpHb to diagnose anemia was 11.3 g/dL for males and 10.2 g/dL for females, with a moderate sensitivity of 83.3% among males and a low 62.9% sensitivity among females. This sensitivity is acceptable for males but considered low for females. However, the specificity for both males and females were at 81% and 91.3%, respectively, which supports the SpHb role to rule out anemia among both. However, males can only be screened for anemia based on their sensitivity results. Khalafallah’s RCT [9] also supports our findings and their results demonstrated that SpHb were more sensitive among males than females.

In another study, by Hornedo-González et al., the negative prediction value (NPV) was greater than 95% for anemia identification, which suggests the ability of the device to rule out the presence of preoperative anemia [30].

### 4.4. PI Effect on SpHb

Park et al. studied the relationship between SpHb and the Perfusion Index. They reported that the Pearson correlation coefficient between the SpHb–Hb bias and the PI was not statistically significant [38]; however, they noticed that the PI values increased after general anesthesia induction, in association with a significant change in the Hb bias from −2.8 to −0.7. Another Australian study, by Khalafallah et al. (2015), noticed that PI values could significantly affect the readings of SpHb, particularly in patients suffering from Raynaud’s disease or other clinical conditions with poor peripheral circulation [9]. They noted significantly lower PI values among females 4.93 (3.66) than males 6.32 (6.57) in their study. These low PI values significantly affected the SpHb–Hb bias among females. In our current study, the mean (SD) of the PI values for males and females collectively was 5.46 (7.03), and in contrast, there was no significant correlation or effect on the SpHb–Hb bias values.

### 4.5. Oxygen Effect on SpHb

Previous reports discussed the relationship between the oxygenation of patients and the accuracy of the optical sensors of various devices. In a study by Gayat et al. (2017), the authors advised that repeated measurements of SpHb should be performed at constant and similar inspired oxygen levels (FiO_2_) [31]. In their study, the Pronto-7 monitor utilizing the Masimo technology was least affected, while the NBM-200 MP monitor (OrSense Ltd., Petah-Tikva, Israel) was significantly impaired. Interpretation of non-invasive Hb readings during bleeding or blood transfusion should be performed at equal FiO_2_ [39,40,41].

## 5. Limitations of This Study

This study focused on only one device and the results can only be applicable to this device. Other studies are also needed to evaluate the accuracy of the spectrophotometric non-invasive hemoglobin measuring method among other devices in the market. The study design focused on a single measurement time, with no continuous measuring points.

The study sample was of limited size and the variations in patients’ demographics, race, and clinical features were not taken into consideration, which may influence the device performance. Race diversity may affect results, as different races are not the same in cutaneous light absorption essential for the spectrophotometric method of hemoglobin measurement [42].

## 6. Conclusions

Non-invasive SpHb concentration underestimated laboratory Hb, with a bias mean difference (SpHb–Hb) of −1.21 g/dL, (SD 1.76). This bias was inversely correlated with laboratory Hb concentrations. SpHb correlated and agreed with laboratory Hb with a good degree of reliability. The identified SpHb cut-off values for anemia were 11.3 g/dL and 10.2 g/dL for males and females, respectively, with a sensitivity of 83.3% for males, but only 62.9% for females. Instant SpHb measurement precision was lower than expected. However, the specificity for both males and females were 81% and 91.3%, respectively. Based on these findings, SpHb can rule out anemia among both males and females, but only diagnose anemia among males. SpHb as a non-invasive screening tool in pre-anesthesia clinics will help exclude the presence of anemia and serves primarily as a time-efficient screening technique to promptly identify anemic patients who may require further therapeutic or diagnostic intervention. This will also reduce the need to recall anemic patients back to clinics when laboratory tests become available. This non-invasive method of measuring hemoglobin (SpHb) still cannot supplant the traditional laboratory Hb tests, which continue to serve as the gold standard for measuring hemoglobin concentrations in blood and the guide for standard health care.

## Figures and Tables

**Table 1:** Demographic and laboratory data that include spectrophotometric hemoglobin (SpHb, g/dL), laboratory hemoglobin (Hb, g/dL), Pleth Variability Index (PVI, %), and Perfusion Index (PI) of studied patients.

Variable		*n* (%)	
Gender	Male	96 (62.2)	
	Female	58 (38.4)	
American Society of Anesthesiology (ASA) Classification	1	55 (36.4)	
	2	65 (43.04)	
	3	31 (20.53)	
	Median	Mean	SD
Age (years)	40	41.29	15.67
BMI (kg/m^2^)	28	28.50	5.09
SpHb (g/dL)	11.2	11.43	2.02
Hb (g/dL)	12.7	12.65	2.30
PVI (%)	13	13.40	5.77
PI	5	5.46	7.03
Oxygen Saturation (%)	99	98.81	1.15

**Table 2:** CBC: laboratory complete blood count; Hb: hemoglobin concentration as measured by complete blood count; SpHb: non-invasive hemoglobin concentration cut-off point; Hb: hemoglobin concentration; CBC: complete blood count; NPV: negative predictive value; PPV: positive predictive value; AUC: area under curve; WHO definitions for anemia: World Health Organization criteria for anemia (Hb < 12 g/dL in females (F) and Hb < 13.0 g/dL in males (M)).

Hb (g/dL)	SpHb	Sens	Spec	NPV	PPV	AUC
WHO (F) Hb < 12	10.2	62.9	91.3	68.4	73	0.775
WHO (M) Hb < 13	11.3	83.3	81	73.9	76.1	0.844
CBC (F + M) Hb < 12	12.9	62.9	52.2	76.8	51.5	0.54
CBC (F + M) Hb < 13	11.3	83.3	81	84.3	78.9	0.88

## Data Availability

Data are available from the authors upon reasonable request.
